# Significant Amplification of Instantaneous Extreme Precipitation With Convective Self‐Aggregation

**DOI:** 10.1029/2021MS002607

**Published:** 2021-11-18

**Authors:** Nicolas A. Da Silva, Caroline Muller, Sara Shamekh, Benjamin Fildier

**Affiliations:** ^1^ Complexity and Climate, Leibniz Centre for Tropical Marine Research Bremen Germany; ^2^ Laboratoire de Météorologie Dynamique (LMD)/Institut Pierre Simon Laplace (IPSL) École Normale Supérieure Paris Sciences & Lettres (PSL) Research University Sorbonne Université École Polytechnique CNRS Paris France; ^3^ Columbia University New York NY USA

**Keywords:** self‐aggregation, convection, precipitation extremes, microphysics

## Abstract

This work explores the effect of convective self‐aggregation on extreme rainfall intensities through an analysis at several stages of the cloud lifecycle. In addition to increases in 3‐hourly extremes consistent with previous studies, we find that instantaneous rainrates increase significantly (+30%). We mainly focus on instantaneous extremes and, using a recent framework, relate their increase to increased precipitation efficiency: the local increase in relative humidity drives larger accretion efficiency and lower re‐evaporation. An in‐depth analysis based on an adapted scaling for precipitation extremes reveals that the dynamic contribution decreases (−25%) while the thermodynamic is slightly enhanced (+5%) with convective self‐aggregation, leading to lower condensation rates. When the atmosphere is more organized into a moist convecting region and a dry convection‐free region, deep convective updrafts are surrounded by a warmer environment which reduces convective instability and thus the dynamic contribution. The moister boundary‐layer explains the positive thermodynamic contribution. The microphysic contribution is increased by +50% with aggregation. The latter is partly due to reduced evaporation of rain falling through a moister near‐cloud environment, but also to the associated larger accretion efficiency. Thus, a potential change in convective organization regimes in a warming climate could lead to an evolution of tropical precipitation extremes significantly different than that expected from thermodynamical considerations. The relevance of self‐aggregation to the real tropics is still debated. Improved fundamental understanding of self‐aggregation, its sensitivity to warming and connection to precipitation extremes, is hence crucial to achieve accurate rainfall projections in a warming climate.

## Key Points

- Convective self‐aggregation may increase both accumulated and instantaneous rainfall extremes
- This increased precipitation is related to reduced rain re‐evaporation and enhanced accretion efficiency with convective self‐aggregation
- Extreme convective updrafts within self‐aggregated convection are weaker due to warmer environment

## 1 Introduction

A large uncertainty regarding tropical precipitation and its evolution in a warming climate is related to convective organization and its response to warming. The spatial organization of deep convection at mesoscales, that is, hundreds of kilometers, is ubiquitous in the tropics in the form of Mesoscale Convective Systems embedded in larger scale perturbations such as the Madden‐Julian Oscillation (Jiang et al., 2020). Convective organization has important societal impacts, notably as it is associated with extreme precipitation (Rossow et al., 2013). A recent observational study (Tan et al., 2015) finds that recent trends in tropical precipitation can be linked to changes in the frequency of occurrence of organized mesoscale cloud systems. Here we will briefly mention the different modes of organization and review the mechanisms involved in the amplification of heavy rainfall with warming—with and without organization—, before focusing on an highly idealized case of organization in this study, convective self‐aggregation.

Mesoscale convective organization can be forced by dynamical processes on large scales (namely, scales similar or larger than mesoscales), for instance vertical wind shear or land‐ocean contrasts and can take the form of mesoscale convective systems or squall lines. It can also arise from internal feedbacks linked to the interaction of clouds with their environment, in the form of large and moist convective areas surrounded by dry subsiding areas (Bretherton et al., 2005; Muller & Held, 2012). In the latter case, spontaneous inhomogeneous spatial clustering of clouds is triggered in otherwise homogeneous unforced environments, a process called self‐aggregation of convection, mainly diagnosed in models so far (Wing et al., 2017). It is important to note that this “clean” mode of organization (in large environments deprived from wind shear, rotation and sea‐surface temperature (SST) gradients) can be far from most situations currently observable, but such a self‐aggregated state is argued to be an alternate stable state of radiative‐convective equilibrium (RCE) that could be reached by the tropics as a whole in a warmer future (Emanuel et al., 2014). Links were also made between some of the processes involved in self‐aggregation simulations and those observed in the real world (Holloway et al., 2017). A natural question that we address here is then: how could precipitation extremes be impacted by self‐aggregation, and why? We will address it using idealized cloud‐resolving simulations run in non‐rotating RCE, and in the absence of wind shear. This “clean” modeling setup will allow a robust attribution of the processes involved, in order to serve as a starting point for integrating more complexity and realism in future modeling experiments.

Several studies have investigated the response of precipitation extremes to warming using idealized simulations of disorganized convection. Two different cloud‐resolving models (CRMs) showed increases in precipitation extremes close to low‐tropospheric moisture (Muller et al., 2011; Romps, 2011). The fact that precipitation extremes follow low‐tropospheric humidity and not column‐integrated humidity can be understood using a theoretical scaling for precipitation extremes, first introduced in Betts (1987) and O'Gorman and Schneider (2009a), and refined to connect it to microphysics (Muller & Takayabu, 2020; Muller et al., 2011). It relates the changes of precipitation extremes to three contributions: a thermodynamic contribution related to water vapor, a dynamic contribution related to vertical mass flux in extreme updrafts, and a microphysic contribution related to precipitation efficiency. The thermodynamic component, dominant in disorganized convection, is not always the prevailing term when convection is organized.

Mechanisms advanced for changes in extremes are sensitive to the mode of organization analyzed. In idealized squall line simulations, Singleton and Toumi (2013) find an amplification of precipitation extremes exceeding significantly the thermodynamic theoretical expectation, when the warming is uniform in height. In the tropics, one expects the atmosphere to warm following a moist adiabat, with larger warming aloft. If instead the warming is uniform with height, a situation which might be more relevant to the mid latitudes, atmospheric instability is enhanced (Loriaux et al., 2013). Consistently, faster updrafts contribute positively to the dynamic contribution, yielding a larger amplification of precipitation extremes with warming than the thermodynamic contribution would entail (Attema et al., 2014; Singleton & Toumi, 2013). We note though that the link between stability and precipitation extremes is not necessarily straightforward. Differences in atmospheric stability may result in differences in convection, but extremes in precipitation intensity do not necessarily follow the atmospheric stability (Hamada et al., 2015).

The increase of precipitation extremes with warming for a given degree of convective organization was also investigated in Muller (2013) but with a warming following the moist adiabat, as expected in tropical atmospheres. In that study, vertical wind shear of varying amplitude is used to organize convection into squall lines. As well as for disorganized convection, precipitation extremes also increase at about 7% $K^{-1}$ of warming for a given degree of convective organization, consistent with the thermodynamic theoretical expectation from the Clausius‐Clapeyron equation (Held & Soden, 2006). However, a change in the degree of convective organization can lead to up to a doubling of extreme rainfall rates. Thus the increase of precipitation extremes from a change in convective organization is larger than that associated with warming by several degrees.

In the case of self‐aggregation, it appears useful to separate the role of warming from the occurrence of aggregation itself when studying the response of extreme rain. Pendergrass et al. (2016) document an SST threshold over which convection self‐aggregates, and the change from unorganized to self‐aggregated convection is associated with an increase of precipitation extremes largely exceeding the Clausius Clapeyron law (super‐CC). Similarly, using CRM experiments, Fildier et al. (2020) also observe a jump in precipitation extremes from disorganized to aggregated simulations. Moreover, they note that super‐CC regimes can occur within aggregated convection but not within the disorganized convection. They link these super‐CC rates to changes in precipitation efficiency. Bao and Sherwood (2019, BS19) also investigate self‐aggregation and precipitation extremes, using a different cloud‐resolving model (WRF). They find that self‐aggregation has a small impact on extreme instantaneous precipitation, but affects strongly daily precipitation extremes. Similarly to Fildier et al. (2020), they note that extreme instantaneous precipitation is more sensitive to microphysical processes. Our work builds on these recent studies, using the theoretical scaling (Muller & Takayabu, 2020) to further investigate and quantify the physical processes associated to the change of precipitation extremes with self‐aggregation. Of particular interest are the following questions:

1. Does convective self‐aggregation only affect rainfall intensities accumulated over time, or also instantaneous rain rates?
2. Which contribution mainly explains the effect of aggregation on extreme rain rates? Is it the thermodynamic, dynamic, or microphysic contribution?
3. Through which physical mechanisms can the change in precipitation efficiency affect rain extremes under convective self‐aggregation?

The next section (§2) describes the cloud‐resolving model and simulations used. Results are presented in §3. The thermodynamic and dynamic contributions to extreme precipitation are analyzed in §4, followed by the microphysic contribution in §5. Conclusions, as well as key implications of our results in a warming climate and outstanding open questions, are discussed in §6.

## 2 Cloud‐Resolving Simulations

### 2.1 Cloud‐Resolving Model

The cloud‐resolving model used here is the System for Atmospheric Modeling, or SAM. This model solves the anelastic conservation equations for momentum, total water and energy. There are six water species in SAM (vapor, liquid cloud water, cloud ice, liquid rain, graupel, snow). The prognostic thermodynamic variables of the model are liquid/ice water static energy, total non‐precipitating water (vapor, liquid cloud water, cloud ice), and total precipitating water (liquid rain, graupel, snow). The longwave and shortwave radiative cooling rates are computed using the radiation code from the National Center for Atmospheric Research (NCAR) Community Atmosphere Model version 3 (CAM3; Collins et al., 2006). As in numerous previous studies of self‐aggregation with SAM, we use the 1‐moment microphysics package. Further details about the model can be found in Khairoutdinov and Randall (2003).

All the runs are in doubly‐periodic geometry starting from homogeneous initial conditions, and with an imposed sea‐surface temperature of 300 K. The resolution is 3 km and the domain size 768 km in both horizontal directions. This relatively small domain size and low resolution do not allow to resolve all convective processes and dynamics and their nonlinear interactions over a wide spectrum range, only from tens to hundreds of kilometers. The vertical grid has 64 levels with the first level at 37.5 m and grid spacing gradually increasing from 80 m near the surface to 400 m above 5 km. To reduce gravity wave reflection and buildup, Newtonian damping is applied to all prognostic variables in the upper third of the model domain (18–27 km altitude).

### 2.2 Simulations

**Figure 1:**
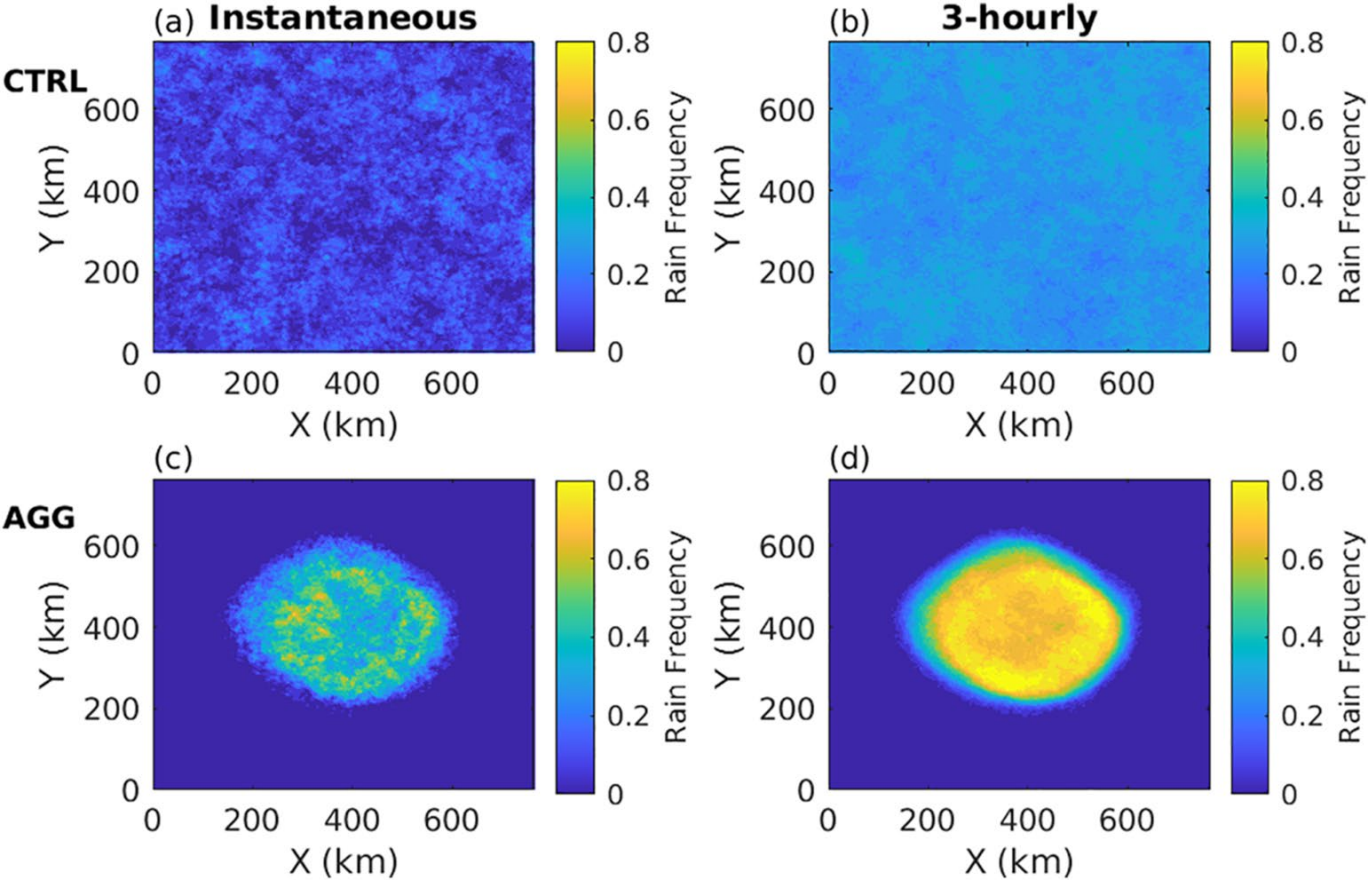
Occurrence frequency of rain over the domain for CTRL (a), (b) and AGG (c), (d) simulations and for both instantaneous (a), (c) and 3‐hourly (b), (d) precipitations.

We perform two simulations. The first (CTRL) with interactive radiative cooling rates (calculated everywhere in the domain at each time step), but these are horizontally homogenized at each time step and height in order to remove radiative feedbacks and thus prevent convective self‐aggregation. In this simulation, convection is somewhat randomly distributed, and precipitating events can occur everywhere in the domain, with convection resembling “pop‐corn” convection (Figures 1a and 1b). Such simulations are occasionally referred to as disorganized RCE.

The second simulation (AGG) has fully‐interactive radiation, namely radiative cooling rates are resolved in 3D space and applied to the temperature tendency at each time step, and self‐aggregates. Conditions favoring convective organization are still a subject of active research, but large domains and the presence of a radiative feedback on the atmospheric circulation are often identified as sufficient conditions for self‐aggregation in models (Muller & Bony, 2015; Muller & Held, 2012). In that case, convection and precipitating events are confined to the moist circular region of the domain (Figures 1c and 1d).

In both cases, the model is run to non‐rotating radiative‐convective equilibrium, or RCE, reached after about 50 days, and the simulations are run for 100 days. Only the last 30 days of the simulations are used for our analysis (day 70 until day 100). Non‐rotating RCE is an idealization of the tropical atmosphere, in which the Earth's rotation is neglected (a reasonable approximation in the tropics where the Coriolis parameter is small), and in which the large‐scale motion (larger than the domain) is neglected. Thus there is no import or export of moist‐static energy into or out of the domain, and in the domain‐mean, the net atmospheric radiative cooling (top of atmosphere minus surface) balances the input of energy into the atmosphere at the surface, namely latent and sensible heat fluxes. The solar irradiance is fixed to ${413\;W.m}^{-2}$ (same as Shamekh et al., 2020), therefore removing diurnal oscillations of convection in the simulations.

## 3 Impact of Convective Aggregation on Precipitation Extremes

### 3.1 Significance for Both Instantaneous and 3‐Hourly Rain

We first investigate the effect of convective aggregation on the frequency of precipitation and on its distribution. The occurrence frequency of both instantaneous and 3‐hourly precipitation over the domain is shown in Figure 1 for both AGG and CTRL simulations. Instantaneous precipitation is not properly ”instantaneous” but refers to an accumulation over a very short time corresponding to the model time step (less than 10 s). Any event is counted as rainy when instantaneous (resp. 3‐hourly) precipitation is strictly positive. Non‐zero thresholds were also tested without giving any significant changes. The CTRL simulation displays frequencies of precipitation rather uniform within the domain, for both instantaneous (Figure 1a) and 3‐hourly precipitation (Figure 1b). As a reference, a perfect white noise sampled over a sufficiently long period of time would be perfectly uniform spatially. Frequencies of precipitation range from 0%–10% for instantaneous precipitation and reach 20%–30% when precipitation is accumulated over 3 h. This increase of precipitation frequency with accumulation time is expected and reflects the increased likelihood of observing precipitation with longer timescales.

The differences are striking when one compares with the frequencies of the AGG simulation (Figures 1c and 1d). While precipitation occurrence displays no particular pattern in the CTRL simulation, rainy events are concentrated on a circular core in the AGG simulation, where frequency of instantaneous precipitation can reach 60%. Conversely, the regions that are far from this circular core, exhibit very low occurrences of precipitation, often even zero (Figure S1 in Supporting Information S1). Because no large‐scale horizontal wind spontaneously develops in the domain, the moist region is almost steady in space, but its size slightly oscillates in time. In both simulations, the spatial distribution of deep convective clouds (and of intense cloud condensation rates) exhibits qualitatively similar patterns than those of precipitation shown in Figure 1 (Figure S2 and S3 in Supporting Information S1).

When one performs a spatial average of the precipitation frequencies over the whole domain, it is found that there are fewer rainy events in the AGG simulation than in the CTRL simulation. Because of the RCE constraint, and similar domain‐averaged radiative cooling rates between the two simulations, mean precipitation only slightly increases from the non‐aggregated to the aggregated simulation (from 3.4 to 3.9 mm/day). Therefore, one could expect a general increase of rainfall rates (when strictly positive) in the AGG simulation compared to the CTRL. In other words, we find that precipitation is less frequent (from a frequency of 7.3%–8.8% between both simulations) but heavier in the AGG simulation, for a similar domain‐mean precipitation.

Differences in the number of rainy events between both simulations are related to the size of the convection core relative to the dry area in the AGG simulation. It is probable that model settings have an influence on the size of the aggregate and thus on rainfall frequencies. For instance, it has been shown that the ability of convection to self‐aggregate in a cloud‐resolving simulation is dependent on the domain size (Jeevanjee & Romps, 2013; Muller & Held, 2012). Nevertheless, we expect that qualitatively, the decreased frequency and increased intensity of precipitation with aggregation is robust.

**Figure 2:**
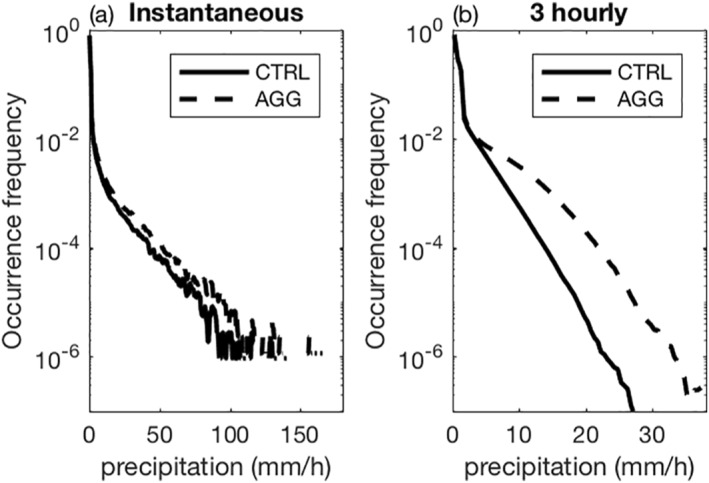
Probability density functions of instantaneous (a) and 3‐hourly (b) precipitation intensity for aggregated (dashed) and unorganized (solid) simulations (Note that only rainy points are included here, which explains the different y‐ranges, as 3‐hourly precipitation is more often non zero than instantaneous precipitation).

We now turn to the full distribution of precipitation in those two simulations. Figure 2 displays the probability density functions (PDF) of instantaneous and 3‐hourly precipitation for both CTRL and AGG simulations. Since our interest lies in precipitation intensity in regions where it rains and its response to aggregation, rather than differences in the area affected by precipitation, we consider the PDF of grid points with strictly positive instantaneous or 3‐hourly precipitation. It corresponds to 7.3% (15.5%) of the total number of grid points in the AGG simulation against 8.8% (29.8%) in the CTRL simulation for instantaneous (3‐hourly) precipitation. Figure 2 shows an increase in the intensity of strong precipitation events, that is, of the high precipitation percentiles, in the AGG simulation. The increase is more pronounced (in relative values) for 3‐hourly precipitation (Figure 2b) than for instantaneous precipitation (Figure 2a). This is consistent with Figure 1 which makes clear that rain accumulates locally over time in AGG, and with BS19 who show that time accumulated (daily in their analysis) precipitation is sensitive to the degree of aggregation. Such hypothesis could be verified using averaged rain intensities on hourly or sub‐hourly outputs and computing the duration of rain events at these high percentiles, unfortunately that is not doable with the present data sampled every three hours.

**Figure 3:**
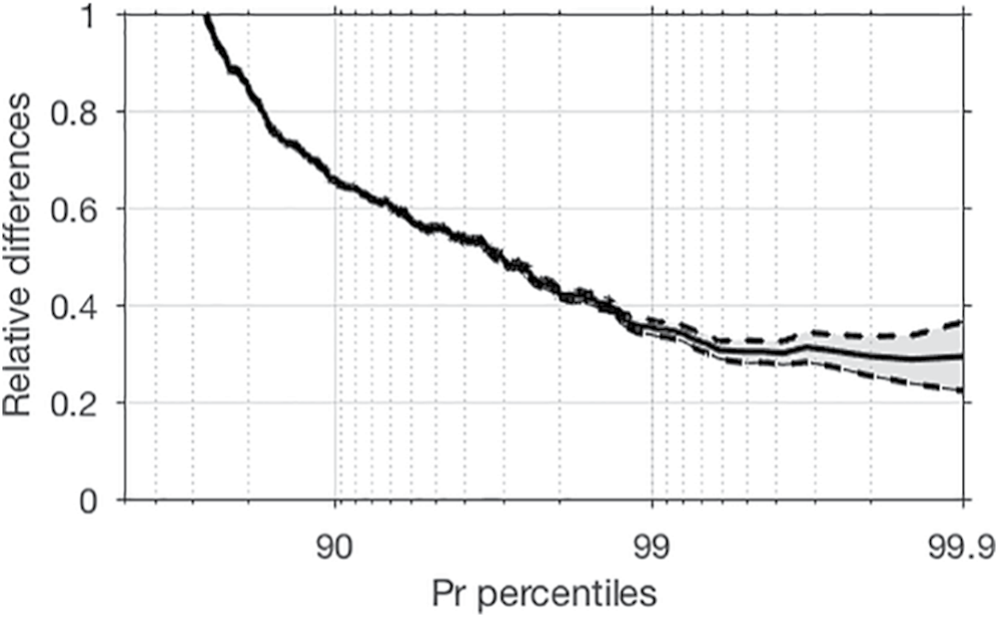
Relative differences of instantaneous precipitation between the aggregated and the unorganized simulation as a function of instantaneous precipitation percentiles for rainy events. Dashed lines and gray shadings correspond to the signal ± one standard deviation.

Unlike the recent study of BS19, we find that instantaneous rainfall rates also increase significantly with aggregation, at a rate of about 30% ± 8% (here ± means one standard deviation for each percentile bin, computed following Fildier et al. (2018), Equation (6) or Appendix C, for estimating the σ‐error on fractional changes between any pairs of simulations) as shown in Figure 3. This figure shows relative differences of instantaneous precipitation as a function of precipitation percentiles. At a fixed percentile rank (e.g., the 99.9th percentile, the threshold in rain intensity for the most intense 1/1000 rainy points), this value increases by 30%, meaning that the most intense 1/1000 rainy points become 30% more intense. As previously, only instants with strictly positive instantaneous precipitation were selected for analysis. Albeit slightly lower, we also find significant increases of instantaneous precipitation extremes in the AGG simulation when including non‐rainy instants (not shown).

The rest of the analysis will thus be devoted to understanding this significant and perhaps surprising increase of instantaneous extreme precipitation in the AGG simulation.

### 3.2 Decomposition Into Thermodynamic, Dynamic, and Microphysic Contribution

The analysis is based on an adaptation of the O'Gorman and Schneider (2009a) scaling of precipitation extremes, which was used in many studies (Loriaux et al., 2017; Muller, 2013; Muller & Takayabu, 2020; Muller et al., 2011):

(1) $$P=\epsilon \int -\rho w\frac{dq_{sat}}{dz}dz=\underset{\text{scaling}}{\underset{︸}{\;\;\epsilon \;\;C\;\;}},$$

where ϵ is precipitation efficiency, ρ is air density, w is vertical velocity, $q_{sat}$ is the saturated water vapor mixing ratio, the derivative is following a moist‐adiabatic parcel ascent, and the integral is performed over positive vertical velocities, thus is a proxy of (gross) condensation rate (which will be replaced by C from now). In the tropics, the atmosphere is close to a moist adiabat and the parcel moves along the vertical to first order, therefore in practice, we used the local derivative

$$\frac{\partial q}{\partial z}$$

as an approximation for the derivative following a parcel ascent. This scaling can be interpreted from a water budget perspective, where a fraction ϵ of the total condensation rate C reaches the ground as surface precipitation P. The remaining fraction of condensates either accumulates as clouds in the column, is advected away, or evaporates either as cloud or precipitation before reaching the ground. This scaling allows to distinguish the contributions to the fractional change in rain intensities, at any percentile, between the CTRL and AGG simulations:

(2) $$\frac{\Delta P}{P}=\underset{\text{microphysic}}{\underset{︸}{\frac{\Delta \epsilon }{\epsilon }}}+\frac{\Delta C}{C},$$

where Δ denotes difference between AGG and CTRL (AGG minus CTRL) and denominators are calculated using the average of the CTRL and AGG values. We now focus on the second term and will investigate microphysical contributions (changes in precipitation efficiency) in Section 5. We first separate the vertical mass flux contribution from the humidity contribution by neglecting second order terms:

(3) $$\frac{\Delta C}{C}\approx \underset{\text{dynamic}}{\underset{︸}{\frac{\int {\left(\frac{-dq_{sat}}{dz}\right)}_{CTRL}\Delta \rho w\;dz}{C}}}+\underset{\text{thermodynamic}}{\underset{︸}{\frac{\int {\left(\rho w\right)}_{CTRL}\Delta \frac{-dq_{sat}}{dz}\;dz}{C}}}$$

The first term of the right‐hand side is the change of condensation rate due to dynamic changes and the second term is the change of condensation rate due to thermodynamic changes.

This equation associates extreme precipitation values with a vertical profile of vertical velocities and saturation mixing ratio. Both of these profiles can be significantly modified with moist convection, and particularly through the formation of precipitation. Indeed, precipitation evaporation has a cooling effect which can both create downdrafts and reduce saturated mixing ratio. It is therefore not obvious that the vertical profile of vertical velocities and the one of the saturated mixing ratios when there is an extreme of surface precipitation, is representative of the earlier processes that led to this extreme.

**Figure 4:**
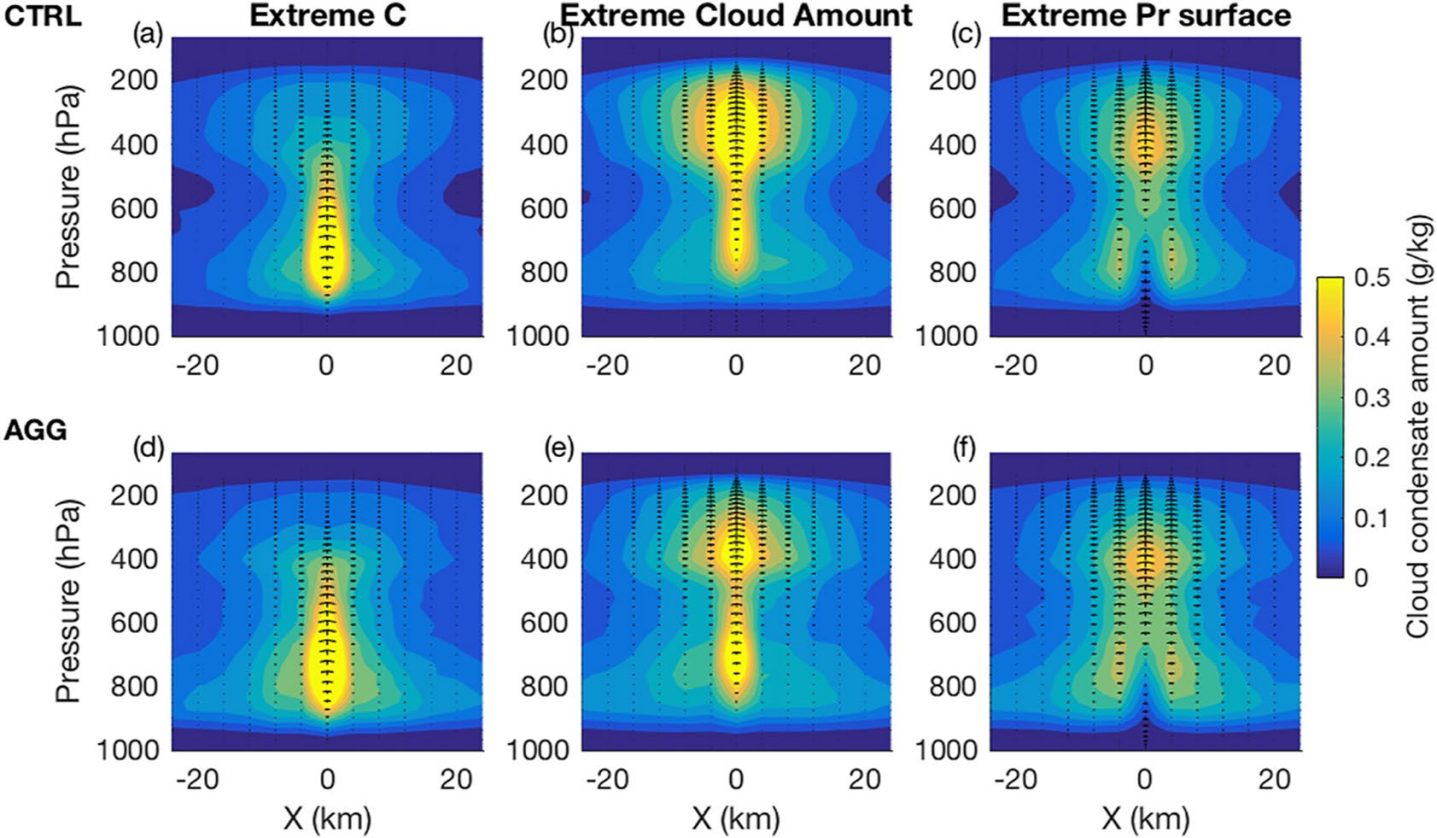
Composites of cloud condensate mixing ratio sections for extremes condensation rates (C; (a), (d)), cloud condensate mixing ratio (b), (e), and surface precipitation (Pr; (c), (f)) for the aggregated (a), (b), (c) and unorganized (d), (e), (f) simulations. Extremes are defined by events above the 99th percentile. Vertical arrows shows vertical velocity. The horizontal axis represents the distance from the grid point that experienced the extreme, and the vertical axis is in pressure coordinates.

Figure 4 displays composites of cloud condensate mixing ratio around points of extreme condensation rate, extreme cloud condensate mixing ratio (both vertically integrated), and extreme surface precipitation for both simulations. If one supposes that these three extremes correspond to a particular time of a same extreme precipitation event, this figure illustrates the changes of clouds properties in between the time of these extremes. In fact, those composites are indeed consistent with the classical cloud lifecycle. First, concomitant with extreme condensation rate, upward motion is initiated in the low troposphere (Figures 4a and 4d). Second, concomitant with extreme cloud amount, vertical updrafts reach the equilibrium level and cloud droplets grow in size and start to precipitate (Figures 4b and 4e). Third, concomitant with extreme surface precipitation, latent cooling from rain evaporation generates intense downdrafts indicated by descending arrows and a higher cloud base above the extreme of surface precipitation (Figures 4c and 4f). Extreme condensation rates were found in only 16% (CTRL) and 27% (AGG) of the clouds at this last stage (Figure S4 in Supporting Information S1). To first order, the cloud lifecycle is well reproduced and visually similar in both simulations. Slight differences can be seen: in the aggregated case, condensate mixing ratios span a larger vertical range in the earlier stage of the lifecycle and a smaller vertical range later on; and vertical velocities are smaller in earlier stages and larger later, suggesting again that extreme rain events may last longer with aggregation. We note that this seems to appear when comparing composites, but individual events can strongly vary around the mean (Figure S5 and S6 in Supporting Information S1).

When evaluating the different contributions of the scaling in Equations 2 and 3, the time chosen to evaluate variables that correspond to extreme surface precipitation is therefore important to have the best estimation for the scaling of precipitation extremes. The vertical velocity of extreme condensation prior to precipitation, is for instance the most relevant to extreme surface precipitation, as it is the effective upward motion that created the extreme of precipitation and which happened earlier in the cloud lifecycle. In the following, condensation rate, thermodynamic and dynamic contributions will be calculated at the extreme of condensation rate, judged more representative of the peak of vertical velocity and condensation preceding extreme precipitation (Section 4). Microphysic contributions will be calculated introducing extreme conversion rates (Section 5) and diagnosed using variables calculated at extreme precipitation (e.g., for rain evaporation; Section 5.1) and at extreme cloud amount (e.g., for autoconversion and accretion in evaporation; Section 5.2).

## 4 Thermodynamic and Dynamic Contributions

**Figure 5:**
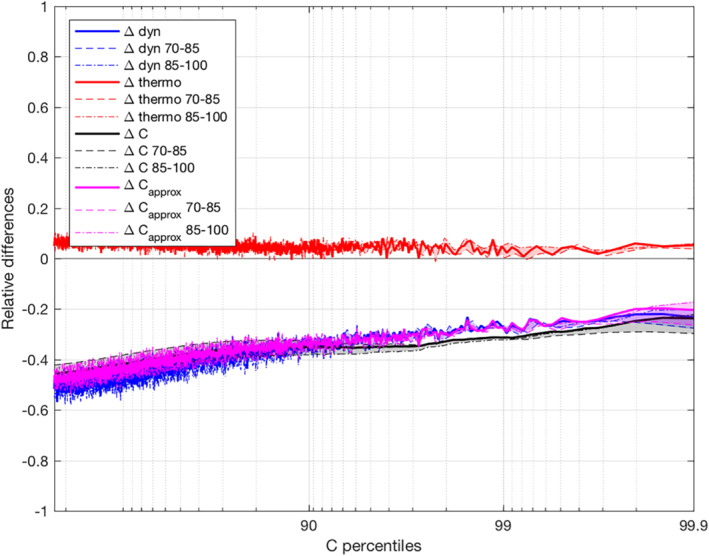
Relative differences of condensation rate (ΔC, full black line, Equation 1) between the AGG and the CTRL simulations as well as their dynamic (Δρw, full blue line) and thermodynamic (Δ∂q/∂z, full red line) contributions as a function of condensation rate percentiles (both computed following Equation 3). Dashed lines correspond to relative differences calculated between the 70th and 85th days whereas dot dashed lines correspond to relative differences calculated between the 85th and the 100th day. The magenta lines are the relative differences of condensation rate calculated as the integral of the product of mean (in each percentile bin) dynamical times mean thermodynamical terms ($\Delta C_{approx}$, unlike ΔC obtained from the mean of the product).

This section investigates the contribution of the changes in condensation rates (ΔC/C in Equation 2) to the changes in precipitation extremes seen in the previous section (ΔP/P in Equation 2) between self‐aggregated and unorganized convection. Figure 5 displays relative differences of condensation rate between the AGG and the CTRL simulations as well as its thermodynamic and dynamic contributions as a function of condensation rate percentiles. Since we selected only the rainy instants to assess the differences of extreme precipitation, we now select the cloudy instants (defined as instants for which the vertically integrated cloud mixing ratio is strictly positive) for assessing the contributions from both the thermodynamic and dynamic. These curves are calculated using mean of variables over 100 samples centered on one condensation rate percentile.

For the calculation of condensation rates (see Equation 1), instead of computing the integral of the mean of the products (Figure 5 in black), one could have computed the integral of the product of the means as represented in magenta in Figure 5. While the former shows the actual condensation rate composited across all events, the latter is more adapted for comparison with the thermodynamic and dynamic contributions. This idea is similar to Fildier et al. (2018), showing that on the scale of a GCM grid box, the scaling in Equation 1 can be computed equivalently before or after compositing on extreme rainfall events. Here, on convective scales, we see on Figure 5 that both calculations give similar relative differences, indicating that non‐linear terms do not act much, and thus validating our separation between dynamic and thermodynamic contributions.

Relative differences have been calculated over all the 30 days but also separately over the first 15 days and the last 15 days to evaluate the uncertainty (gray shadings in Figure 5). It shows small changes between the first 15 and last 15 days for both contributions, giving us confidence that extremes have converged and that results are robust.

**Figure 6:**
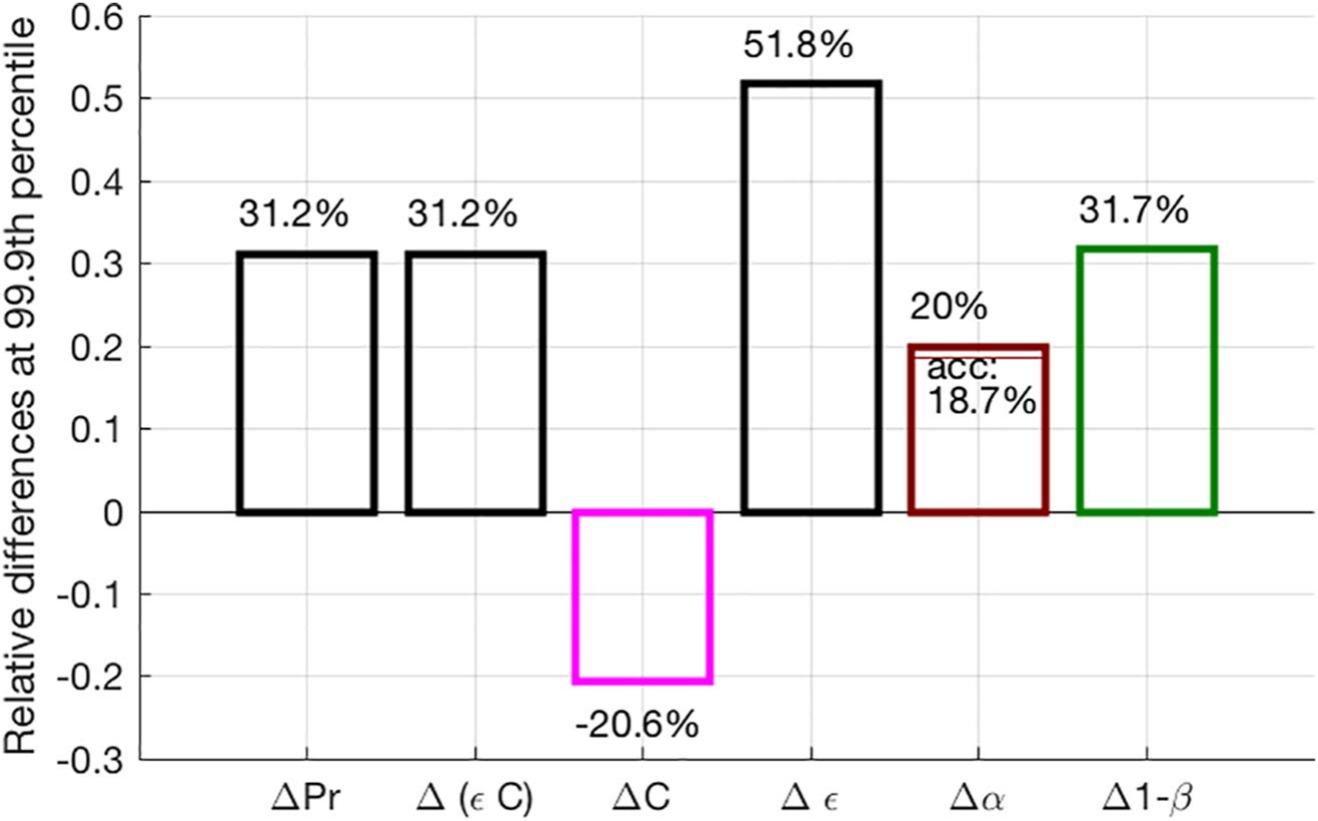
Relative differences at the 99.9th percentile of: instantaneous precipitation from a direct calculation (ΔPr) and from the scaling used in Equation 3 (Δ(ϵC)), condensation rates (ΔC), precipitation efficiency (Δϵ), conversion efficiency (Δα) with the contribution from accretion efficiency, and sedimentation efficiency (Δ1−β).

One can see a decrease of about −20 % in extreme condensation rate in the AGG simulation compared to the CTRL simulation. This negative contribution is also shown in Figure 6, which summarizes the main contributions to the changes in precipitation extremes between both simulations according to Equation 3. The decrease in extreme condensation rate is explained by a decreased dynamic contribution (−25%) in the AGG simulation, while the thermodynamic contribution remains similar or even slightly higher in the AGG simulation (+5%).

### 4.1 Thermodynamic Contribution Driven by Surface Humidity Changes

**Figure 7:**
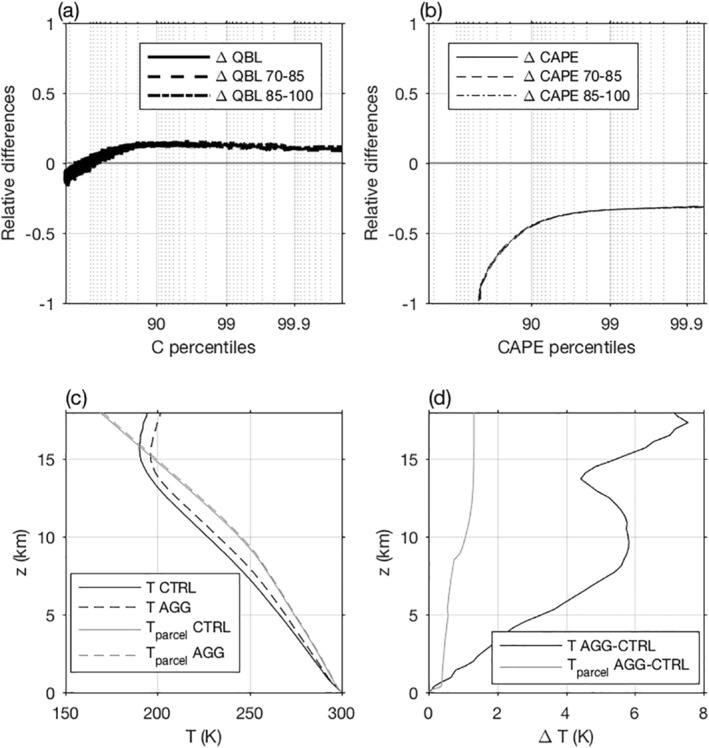
Relative differences in boundary layer water vapor mixing ratio (QBL, (a)) and Convective Available Potential Energy (CAPE, (b)) between the aggregated and the unorganized simulations as a function of condensation rate (C) and CAPE percentiles (resp.). Dashed lines correspond to relative differences calculated between the 70th and 85th days whereas dot dashed lines correspond to relative differences calculated between the 85th and the 100th day. Environmental and parcel vertical profiles of temperature for the aggregated and unorganized simulations (c) and differences of these profiles between both simulations (d), for events above the 99.9th percentile of CAPE.

In order to explain positive and negative contributions of the thermodynamics and dynamics, changes in boundary layer water vapor mixing ratio (QBL) and Convective Available Potential Energy (CAPE) are displayed as a function of C and CAPE percentiles in Figures 7a and 7b. Indeed, the former is related to the thermodynamic contribution, and the latter to the dynamic contribution (Muller & Takayabu, 2020). For consistency with the previous analysis, QBL was calculated only for cloudy instants.

One can observe an increase of about +10% of QBL in the AGG simulation for extreme C. This is qualitatively consistent, though slightly larger, than the thermodynamic contribution. A positive thermodynamic contribution is expected since self‐aggregation is associated with the confinement, and thus increase, of moisture in the convective region (Figure 1). This increase in QBL is also consistent with a warming of a few degrees with aggregation if we assume the boundary layer saturated (which is a reasonable assumption in regions with high condensation rates).

As precised in Betts (1987); O'Gorman and Schneider (2009a), the moist‐adiabatic derivative of saturation specific humidity does not increase (in absolute value) as quickly as does the saturated specific humidity with temperature. This may explain the higher rate of QBL changes compared to the thermodynamic contribution. This observation implies that in the process of cloud formation, part of the water vapor raised from the boundary layer remains not condensed at the top of the cloud, and particularly in the AGG simulation which is warmer and exhibits lower cloud tops than in the unorganized simulation (Figures 7c and 7d).

Another potential reason explaining the discrepancy between the QBL relative differences and the value of the thermodynamic contribution is the limit of our method. Indeed, at the time of extreme C, QBL may not be representative of water vapor mixing ratio used for condensation which might result in unexplained differences. The scaling itself is also not perfect, local vertical variation of water vapor mixing ratio is only an approximation of the variation of water vapor mixing ratio that a parcel would experience when being raised: horizontal advection and losses by entrainment are not taken into account in this simplified scaling. Yet, we believe that the more humid boundary layer with aggregation is responsible for the increased thermodynamic contribution.

### 4.2 Dynamic Contribution Driven by Changes in Local CAPE

Changes of CAPE between each simulation are shown as a function of CAPE percentiles since part of the CAPE is already consumed at extreme C, although this results in only small differences between each simulation at extreme C (not shown). Only strictly positive CAPE events were selected for analysis, in order to avoid the dry region of the AGG simulation (as done previously for precipitation, C, and QBL). At extreme CAPE, there is a decrease of about −30% of the CAPE in the AGG simulation, which would be consistent with a −15% decrease in extreme vertical velocities (proportional to the square root of CAPE). This value is thus smaller but qualitatively consistent with the dynamic contribution decrease of −25%. With the use of similar idealized simulations, Windmiller and Hohenegger (2019) showed that in such consecutively active regions, convection is preferentially triggered at the edge, where the CAPE is not maximal. This would imply that retaining the highest CAPE percentiles in the AGG simulation may overestimate the actual energy involved in the formation of extreme precipitation in this simulation, explaining the slight mismatch between the dynamic contribution and the one expected from CAPE differences at extreme CAPE percentiles.

The decrease in CAPE can be understood by considering temperature profiles of a near‐surface parcel rising adiabatically, and of its near‐environment (Figures 7c and 7d). The parcel temperature is somewhat similar with and without aggregation. The environmental temperature profile on the other hand is much warmer in the aggregated simulation. In other words, the atmosphere is closer to a moist adiabat with aggregation. This is expected, as with aggregation, convection and moisture are confined to a long‐lasting moist region. So the entrained air at the edge of clouds is relatively moist, leading to a small net effect of turbulent entrainment at the edge of rising plumes (as the entrained air has properties close to the rising air; Singh and O'Gorman (2015)). Thus the atmospheric temperature is closer to an undilute moist adiabatic profile of the parcel, leading to smaller CAPE.

The extreme condensation rates are thus lower in the AGG simulation because of less favorable atmospheric instability, barely tempered by increased low‐level moisture. These processes occur in the early stages of precipitation extreme formation and are followed by microphysical effects allowing the evolution of cloud drops to precipitating drops reaching the surface. The contribution of these effects to the enhanced precipitation extremes in the AGG simulation are discussed in the next section.

## 5 Microphysic Contribution

**Figure 8:**
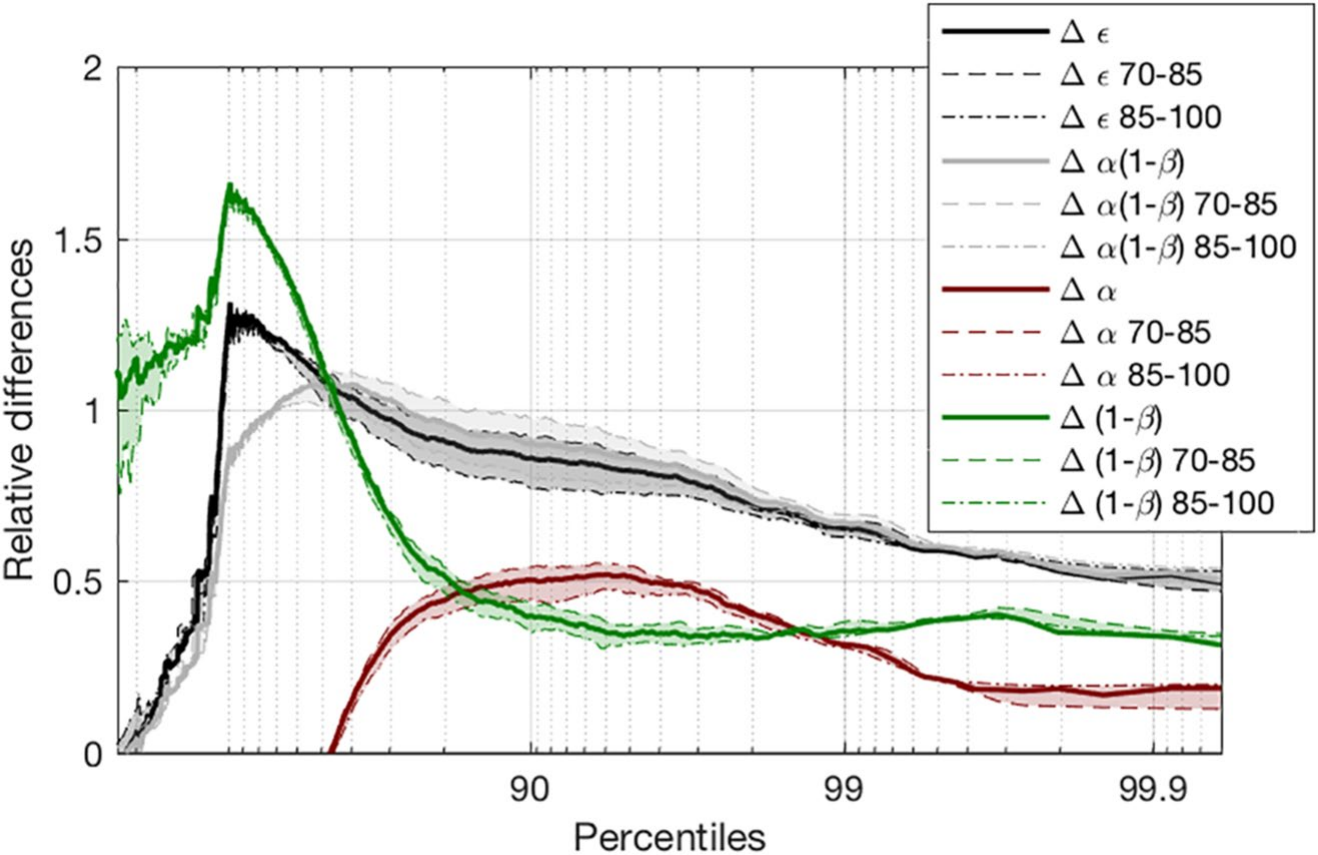
Relative differences of precipitation efficiency (Δϵ, full black line) between the AGG and the CTRL simulations as well as their contribution from the conversion of precipitation (Δα, full dark red line) and from the evaporation of precipitation (Δβ, full green line) as a function of surface precipitation percentiles. Dashed lines correspond to relative differences calculated between the 70th and 85th days whereas dot dashed lines correspond to relative differences calculated between the 85th and the 100th day.

This section investigates the contribution of the changes in precipitation efficiency (Δϵ/ϵ in Equation 2) to the changes in precipitation extremes seen in Section 3 (ΔP/P in Equation 2) between self‐aggregated and unorganized convection. Figure 8 shows relative differences of precipitation efficiency between the AGG and the CTRL simulation as function of precipitation percentiles. Precipitation efficiencies were calculated dividing precipitation by a corresponding condensation rate. More precisely, bins of 100 samples were done for precipitation keeping only precipitation events and for condensation rates keeping only cloudy events. Both precipitation and condensation rate bins were classified in ascending order of precipitation or condensation rate (respectively). Since some clouds do not precipitate, the number of bins of condensation rate is higher than the one of precipitation. Therefore, the lowest condensate rate bins were removed and considered to not produce precipitation. It resulted in an equal number of bins for precipitation and condensation rate. Precipitation efficiencies were calculated dividing the mean of each precipitation bin by the mean of the corresponding condensation rate bin (i.e., percentile by percentile).

The precipitation efficiency is found to be the largest contribution to the change of precipitation extremes between both simulations, with an increase of +50% in the AGG simulation (Figures 6 and 8). The values of precipitation efficiency at the 99.9^th^ percentile reach about 0.48 in the AGG simulation against 0.28 in the CTRL simulation (Figure S7 in Supporting Information S1; we note in passing that these values are within the range of existing estimates in the literature for similar simulations (Fildier et al., 2020; Lutsko & Cronin, 2018), though the precise values are somewhat sensitive to the microphysics and to the percentile of precipitation considered). In order to further investigate this change in precipitation efficiency, we follow Lutsko and Cronin (2018) and split the precipitation efficiency ϵ into a term involving cloud to rain conversion efficiency α, and a term involving rain to surface precipitation efficiency (1−β):

(4) $$\epsilon \;\;=\;\;\frac{P}{C}\;\;=\;\;\frac{\int \rho q_{p}^{src}dz}{C}\;\;\times \;\;\frac{P}{\int \rho q_{p}^{src}dz}\;\;=\;\;\alpha \times \left(1-\beta \right),$$

where $q_{p}^{src}$ is the source (in kg ${kg}^{-1}\;s^{-1}$) of precipitating condensate amount $q_{p}$ from the microphysics, and is directly diagnosed in the model outputs.

In other words, α is the rate of conversion from cloud to precipitating condensate $q_{p}^{src}$, normalized by the rate of conversion from water vapor to cloud condensate C. Therefore, α is called conversion efficiency (Lutsko & Cronin, 2018). The conversion efficiency α increases by about +20% with aggregation, for extreme precipitation events (Figures 6 and 8). The extremes of $q_{p}^{src}$ were evaluated at $q_{p}^{src}$ percentiles to reflect the intermediate stage between the maximum in cloud condensation and the maximum in surface precipitation reached in a typical cloud lifecycle.

The other term, (1−β), referred as sedimentation efficiency (Lutsko & Cronin, 2018), represents the fraction of source of precipitating condensate from microphysics which reaches the ground as surface precipitation. This fraction is typically less than unity because of rain evaporation as precipitating condensates falls through subsaturated air. The increased sedimentation efficiency clearly dominates the precipitation efficiency increase, contributing approximately to +30% (Figures 6 and 8).

In the next sections we investigate these changes of sedimentation (1−β) and conversion (α) efficiency between both simulations in more details.

### 5.1 Changes in Rain Evaporation From Changes in Saturation Deficit

The term 1−β is influenced by precipitation evaporation and precipitation transport (detrainment or entrainment). In the following, we assume that precipitation evaporation is the leading factor explaining the ratio 1−β.

**Figure 9:**
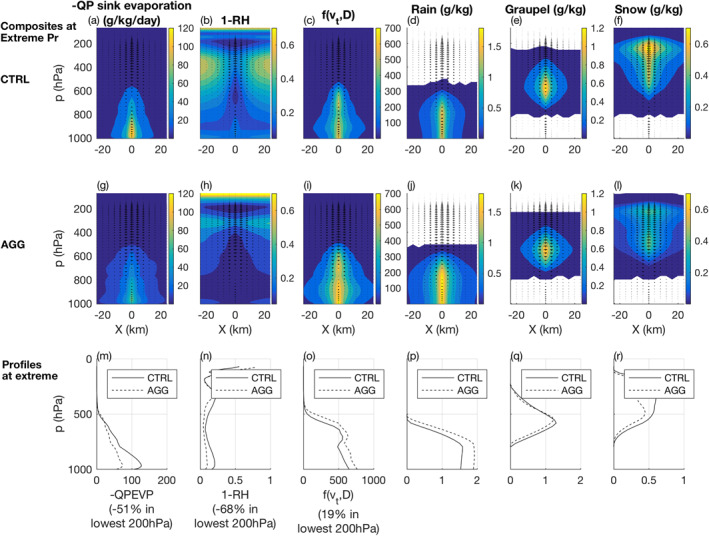
Composites of (a), (g), (m) rain evaporation, (b), (h), (n) distance to saturation, (c), (i), (o) ventilation function $f\left(v_{t},D\right)=aD+bD\sqrt{Dv_{t}}$, (d), (j), (p) rain mixing ratio, (e), (l), (q) graupel mixing ratio, (f), (l), (r) snow mixing ratio at times of extreme surface precipitation. The top panels show the CTRL simulation and the middle panels the AGG simulation in the vicinity of the extreme precipitation event (located at X=0). The bottom panels show the profiles at the extreme precipitation location (i.e., at X=0).

In SAM, the rate of precipitation evaporation

$$-{\frac{\partial q_{p}}{\partial t}}_{evap}\propto \left(1-RH\right)f\left(v_{t},\;D\right)$$

is proportional to the distance to saturation 1−RH and to a function $f\left(v_{t},D\right)$ of both the condensate terminal velocity ($v_{t}$) and the mean diameter of precipitation particles (D): $f\left(v_{t},D\right)=aD+bD\sqrt{Dv_{t}}$. Where a and b can be considered as constants in our case. This latter term involves the so‐called ventilation factor and accounts for the surface of precipitation particle available for evaporation. These variables, along with liquid, graupel and snow mixing ratios, are plotted in Figure 9.

It shows that the evaporation of precipitation is less important in the aggregated simulation than in the unorganized simulation. The decrease in precipitation evaporation in the aggregated simulation is particularly significant in the lowest parts of the troposphere, where most of the precipitation evaporation occurs. Indeed, we found a decrease close to −50% in the lowest 200 hPa of the atmosphere in the aggregated case. At these altitudes, the saturation deficit is about 68% less important in the aggregated simulation than in the unorganized simulation. Thus, the moister environment of the aggregated precipitation extremes largely explains the reduced precipitation evaporation near the surface. Finally, increased precipitation terminal velocity and diameter in the aggregated simulation, tends to temper (through $f\left(v_{t},D\right)$) the differences of precipitation evaporation between both simulations by nearly 19% in the lowest 200 hPa of the troposphere (Figures 9c, 9i and 9o).

Thus, consistent with BS19, we also find faster terminal velocities with aggregation, but in our case it is not due to a change in graupel. Instead, it is due to increased rain and decreased snow amounts (consistent with the warmer temperatures), with little change in graupel (Figures 9d–9f). BS19 further note that the faster terminal velocities, and associated shorter residence times, of rain compared to graupel or snow, imply reduced rain evaporation in their simulations. But for instantaneous precipitation rates, we believe that the instantaneous rain evaporation (Figure 9a) is more relevant, which instead increases with terminal velocity (Figures 9c, 9i, and 9o) due to the aforementioned ventilation effect. This effect partially offsets the reduced rain evaporation from increased humidity (Figures 9b, 9h, and 9n).

### 5.2 Larger Conversion Efficiency Due to More Efficient Accretion

**Figure 10:**
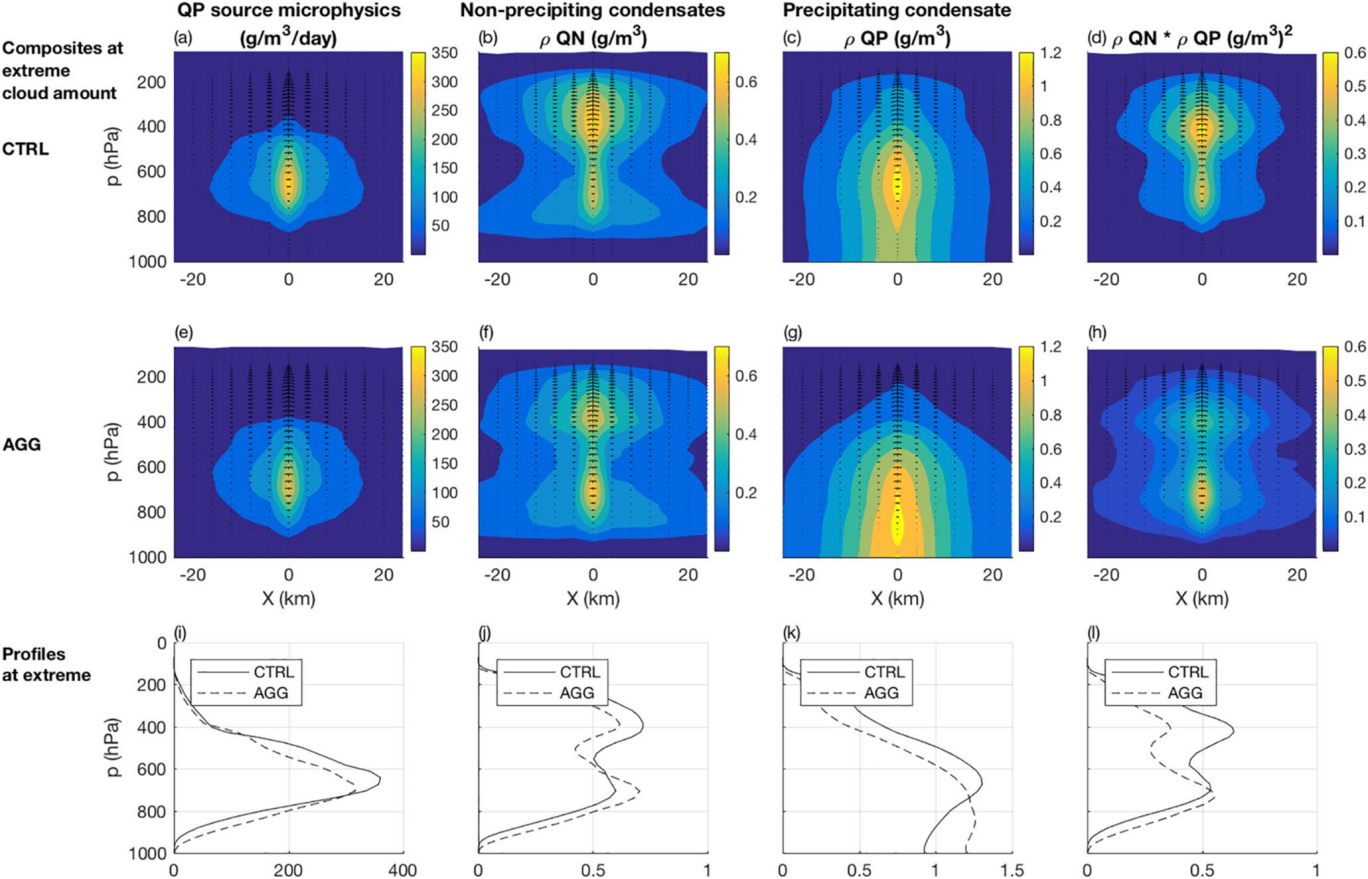
Composites of (a), (e), (i) precipitation source from microphysics, (b), (f), (j) non‐precipitating condensates, (c), (g), (k) precipitating condensates, and (d), (h), (l) ρQN×ρQP at times of extreme cloud amount. The top panels show the CTRL simulation and the middle panels the AGG simulation in the vicinity of the extreme event (located at X=0). The bottom panels show the profiles at the extreme location (i.e., at X=0).

We noted that the conversion efficiency of extreme events α increases by about 20% in the aggregated simulation. Since we also showed that extreme condensation rates decrease by 20% in the aggregated simulation, it implies that the extreme conversion rates remain similar in both simulations. This is indeed what we observe in average when comparing the vertical profiles of extreme conversion rates between both simulations (Figures 10a, 10e and 10i), although the conversion rates are higher near the surface and smaller aloft for the aggregated simulation compared to the unorganized simulation.

In the 1‐moment microphysics scheme that we use (Khairoutdinov & Randall, 2003), conversion from cloud to precipitating condensate is quantified as the sum of two microphysical processes:

• Accretion of precipitating type $q_{p}$ due to collection of cloud condensate $q_{n}$:

$${\left(\frac{\partial q_{p}}{\partial t}\right)}_{acc}\propto q_{n}q_{p}^{a}$$

where the exponent a depends slightly on the precipitation type (rain, graupel or snow in SAM) but is typically close to 1.

• Autoconversion and aggregation of cloud liquid and ice condensates (resp.)

$${\left(\frac{\partial q_{p}}{\partial t}\right)}_{auto}\propto {\left(q_{n}-q_{n,0}\right)}_{+}$$

where the coefficient of proportionality and threshold $q_{n,0}$ are different for cloud liquid and cloud ice, and where subscript + indicates that negative values are replaced by zero.

Decomposing α into contributions from both processes (through the decomposition of $q_{p}^{src}$ into an accretion plus an autoconversion tendency in Equation 4), we see that the increase of α in the aggregated simulation is almost entirely due to the first process, namely accretion, with a contribution of +18.7% among the +20% (Figure 8b). We thus draw the vertical profiles of cloud condensates, precipitation, and their product as an estimation of the accretion rate vertical profiles for both simulations (Figures 10b–10d and 10–10h). The differences between the simulations change sign with altitude. In the highest altitudes (above 700 hPa), there is more cloud condensates and more precipitation in the unorganized simulation, which leads to higher accretion and conversion rates.

This increased condensate load aloft can be attributed to the increased condensation rates in the unorganized simulation. The result is an increase of auto‐conversion rates at first which creates more precipitation particles, which combined with the increased condensate loads, provides a further increase of precipitation conversion through accretion in the unorganized simulation. The situation is different closer to the surface (in the lowest 200 hPa) where one can observe increased cloud condensates and precipitation particles, leading to increased accretion in the aggregated simulation. While we do not have evidence for this, the increased cloud condensates likely comes from the lower cloud bases in the aggregated simulation (a consequence of the increased surface relative humidity) and/or the longer cloud lives in the aggregated simulation (as can be expected from the comparison of 3‐hourly vs. instantaneous precipitation differences in Figure 2). But the main increase of accretion rates near the surface arises from the increased precipitation explained by reduced evaporation in the aggregated simulation (Figures 9a, 9g, and 9m).

These differences between low and high altitudes tend to compensate each other and make averaged extreme accretion rates rather similar between both simulations. The effect is thus an increase of the accretion efficiency α in the aggregated simulation.

## 6 Discussion and Conclusions

The spatial organization of deep‐convective clouds is ubiquitous in the tropics and often leads to extreme weather. Importantly, one must question the extent to which models and idealized experiments can reproduce observed organized features and inform the rain changes that they induce. Two opposite types of organization are often studied in CRMs: regular cloud structures versus convective clusters or moist patches, and metrics exist to test which type is more prevalent (Tompkins & Semie, 2017). Both organization types are sensitive to horizontal mixing in various ways (Craig & Mack, 2013; Piotrowski et al., 2009; Tompkins & Semie, 2017; Windmiller & Craig, 2019) and are controlled by different mechanisms occurring in the real atmosphere. By suppressing numerical dissipation, Piotrowski et al. (2009) observed that an original round feature of organized convection is not maintained, suggesting that some models cannot host convective organization without diffusion (numerical or through the turbulent scheme). Regular organization structures are controlled by cellular convection and cold pool interactions in the boundary layer (Feingold et al., 2010; Haerter, 2019). Instead, clustering, or aggregation, is driven by feedbacks that couple radiative cooling and convection, and wind effects on surface evaporation, leading to atmospheric instabilities in RCE (Bretherton et al., 2005; Emanuel et al., 2014). Here we focus on the latter, in a highly idealized manner. Although idealized self‐aggregation of convection as modeled in square homogeneous RCE domains may be scarcely met in the current tropics, such states may be reached after a certain degree of warming (Wing & Emanuel, 2012) and some links with real world cloud clustering are already observable in current climate (Holloway et al., 2017). The present work does not address this question, but instead undertakes a detailed analysis of the mechanisms through which self‐aggregation could affect precipitation extremes, once it occurs, if it occurs.

To do so, we analyze two idealized simulations using the SAM cloud‐resolving model. In the first simulation, the atmosphere is organized into a moist convecting core and a dry convection‐free region while in the second simulation convection is disorganized. We find that convective aggregation significantly increases precipitation extremes, by 70% for the 99.9th percentile of 3‐hourly precipitation consistent with earlier studies, but also surprisingly by a significant 30% increase for the 99.9th percentile of instantaneous precipitation.

To investigate the processes responsible for the increase of instantaneous precipitation extremes with aggregation, we use an adapted scaling of precipitation extremes which accounts for the lifecycle of deep convective clouds. Previous studies using the scaling of precipitation extremes associated surface precipitation extremes with condensation rates and precipitation efficiencies co‐located in time and space (Loriaux et al., 2017; Muller, 2013; Muller et al., 2011; O'Gorman & Schneider, 2009b). We argue that such spatio‐temporal association may not be the most accurate method for investigating the causes of extreme precipitation, the latter significantly modifying the vertical profiles of thermodynamical and dynamical variables at their onset. Instead, one may evaluate the scaling variables a few minutes or hours before the extreme of precipitation (Fildier et al., 2020, e.g.,). Although this alternative method would likely produce a more accurate relationship between precipitation, precipitation efficiency, and condensation rates, a bulk application is difficult since the location and time of the perfect scaling variables of one extreme precipitation event likely differ between these events. Here, we adopt another approach allowing to address this issue in a bulk manner and consisting in associating extreme precipitation percentiles with extreme percentiles of condensation and conversion rates, which mimicks different stages in the lifecycle of individual deep convective clouds. We use a decomposition of precipitation efficiency in a conversion and an evaporation term (as done in Lutsko and Cronin (2018)) and further investigate the role of the main microphysical variables. It is worth noting that such analyses could be reproduced in many other simulation or observational contexts.

Within this framework, we find that precipitation extremes are mainly increased through larger precipitation efficiency with convective aggregation. The contribution from the dynamics is largely negative with −25% while the thermodynamic contribution accounts for a comparatively small +5% contribution.

The increased thermodynamic contribution comes from enhanced moisture confinement in the boundary layer of the moist region for the case of aggregated convection, enabling convective updrafts to carry more water. The decreased dynamic contribution similarly derives from the moisture confinement in the moist convecting region, which reduces the effect of turbulent entrainment of environmental air at the edge of clouds. This results in an atmosphere closer to a moist adiabat, thereby reducing the strength of convective updrafts through a decrease of their buoyancy. Among these two opposite effects, the dynamical effect is the most important, and causes a 20% reduction of cloud condensation rates in convectively aggregated extreme events.

The microphysic contribution is the leading contribution to precipitation extremes enhancement, with a 50% increase of precipitation efficiency when convection is aggregated. Reduced rain evaporation contributes about 30% because of the moister environment of falling rain with aggregation. This is particularly true in the lowest 200 hPa where we find a 50% reduction of evaporation rates with aggregation. Increased accretion efficiency contributes another 20% increase in aggregated extreme precipitation.

The present results should be interpreted with care. It must be acknowledged that convective aggregation is sensitive to the modeling setup and forcing conditions chosen; this analysis is only one of a few starting points to study the response of extreme rain to changes in the organization of convection. Of particular importance is the sensitivity to the choice of modeling setup and subgrid‐scale parameterizations, increasingly well‐known and documented in the community. For instance, the degree of aggregation can be affected by the domain size and shape (rectangle vs. square, or long‐channel domains; Cronin and Wing (2017)), thus affecting extreme precipitation intensities (Abbott et al., 2020). Coarse resolutions can also reduce the reevaporation of rain in downdrafts: that can inhibit cold pools and thus trigger aggregation (Jeevanjee & Romps, 2013). The representation of low clouds and the degree of convective mixing is also affected by the coarse resolutions and the turbulence scheme (Holloway et al., 2017; Tompkins & Semie, 2017) which affects the strength of aggregation, either through a moisture‐memory feedback (Tompkins & Semie, 2017) or through a reduction in the radiative feedback (Muller & Held, 2012). This non‐exhaustive list of modeling knobs that can trigger or inhibit aggregation is pursued by a whole field of research to further study the conditions in which self‐aggregation can affect the real atmosphere, now and in the future.

Our results are qualitatively consistent with recent results from BS19, in the sense that the microphysics has a leading role in explaining extreme instantaneous precipitation in self‐aggregated simulation. However, their idealized WRF simulations only exhibit a small change of instantaneous precipitation with aggregation. This may reflect the fact that microphysics is not well constrained in observations and models. Therefore, more work is desirable to investigate how precipitation efficiency affects precipitation extremes with convective aggregation, and how sensitive these results may be to the microphysics formulation in models. Three microphysics schemes exist in the System for Atmospheric Modeling used here: a single‐moment scheme developed in the original version of the model and used for our simulations (Khairoutdinov & Randall, 2003), and two double‐moment microphysics schemes from Morrison et al. (2005) and Thompson et al. (2008). Knowing which microphysics scheme is most realistic should also be investigated, although the single‐moment scheme used here has also been used in Global Cloud‐Resolving Model experiments, resulting in model outputs that are visually indistinguishable from satellite observations (Stevens et al., 2019). The use of a spectral bin microphysics scheme would certainly provide a further step towards realism. Despite their high computational cost, such schemes have proven their better accuracy compared to bulk microphysics schemes (Khain et al., 2015). In an interest of making simulations closer to the real atmosphere, many other sensitivity tests must also be performed, by testing other parameterizations and model configurations as mentioned above.

The present work uses the surface‐based CAPE to understand the decreased strength of convective updrafts when convection is aggregated. While we were mostly interested in a diagnosis rather than a precise estimation, it is worth noting that this metric may not be a good representation of every convective potentials. More elaborated metrics may be tested for that purpose such as the Most‐Unstable CAPE, the mixed‐layer CAPE, the generalized CAPE (Steinacker, 2017) or the Potential Energy Convertibility (Yano et al., 2005). The improvement of the metrics allowing to determine the potential intensity of convection is desirable for a better understanding of the changes in precipitation extremes with convective organization.

In a warming climate, one may expect an increase of precipitation extremes according to the Clausius‐Clapeyron law which relates each degree of warming by an increase of about +7% of precipitation extremes. Our results suggest that this increase could be even more dramatic depending on the change of convective organization with temperature. Recent studies investigated the change of convective aggregation with SST under similar idealized settings. It is generally admitted that high SSTs favor convective self‐aggregation (Pendergrass et al., 2016). Shamekh et al. (2020) showed that SST gradients are important as well for triggering convective aggregation. Overall the response of convective aggregation to warming remains uncertain and still an area of active research (Wing, 2019).

## Supporting information

Supporting Information S1

## Data Availability

The simulation data set is freely available at https://figshare.com/s/5ff1811271b1f3643004.
