# Supplementary material for: Significant Amplification of Instantaneous Extreme Precipitation With Convective Self‐Aggregation
Source: J Adv Model Earth Syst. 2021 Nov 18;13(11):e2021MS002607. doi: 10.1029/2021MS002607 (PMC9285386; doi:10.1029/2021MS002607)
Supplement: Supplementary file 1 — Supporting Information S1 [file JAME-13-0-s001.pdf]

Supporting Information for "Significant amplification of instantaneous extreme precipitation with convective self-aggregation"

Nicolas A. Da Silva[1], Caroline Muller[2], Sara Shamekh[3], Benjamin Fildier[2]

[1]Complexity and Climate, Leibniz Centre for Tropical Marine Research, Fahrenheitstrasse 6, 28359, Bremen, Germany

[2]Laboratoire de Météorologie Dynamique (LMD)/Institut Pierre Simon Laplace (IPSL), École Normale Supérieure, Paris Sciences & Lettres (PSL) Research University, Sorbonne Université, École Polytechnique, CNRS, F-75005 Paris, France

[3]Columbia University, New York 10032, USA

Contents of this file

1. Figures S1-S7

Introduction

These additional figures provide the reader a better visualisation of the simulations that were used for analysis in the main text.

Descriptions of the Figures

Figure S1 shows the fraction of pixels experiencing a given rain frequency (in Fig. 1 of the main manuscript) as a function of rain frequency.

Figure S2 displays snapshots of cloud water path at different instants in the AGG simulation.

Figure S3 shows frequency maps of extreme instantaneous precipitation and condensation rates in both AGG and CTRL simulations.

Figure S4 shows the conditional probability of finding an extreme of condensation rate at a given distance from an extreme of surface precipitation. In order to have comparable estimates between different distances, the probabilities (or rather frequencies) were normalized by the number of grid points located at the same distance from the precipitation extreme location.

Figure S5 shows cloud condensate mixing ratio vertical sections for individual extreme condensate rate, cloud amount and precipitation events. These individual events are part of the average made in Fig. 4 in the main manuscript.

Figure S6 shows signal to noise ratio vertical sections of cloud (defined as strictly positive cloud condensate mixing ratio) frequency for extreme condensation rates, cloud amount, and precipitation events.

Figure S7 shows precipitation efficiency in both AGG and CTRL simulations and their relative differences as a function of percentile.

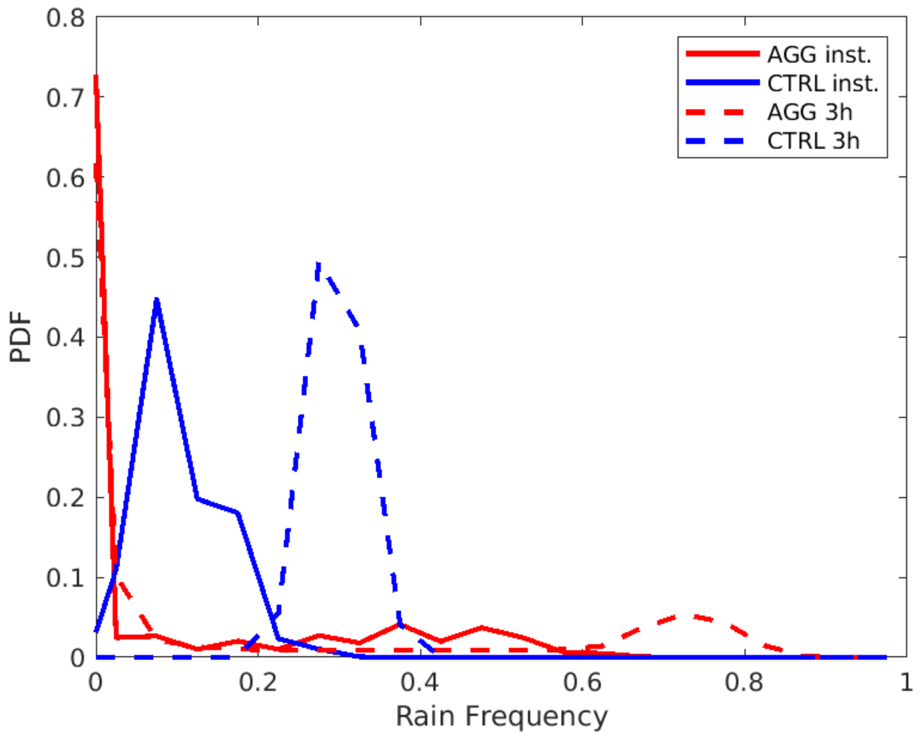

Figure S1. Probability density functions (PDF) of rain frequencies across the domain for instantaneous (plain) and 3-hourly (dashed) rainfall, for both AGG (red) and CTRL (blue) simulations.

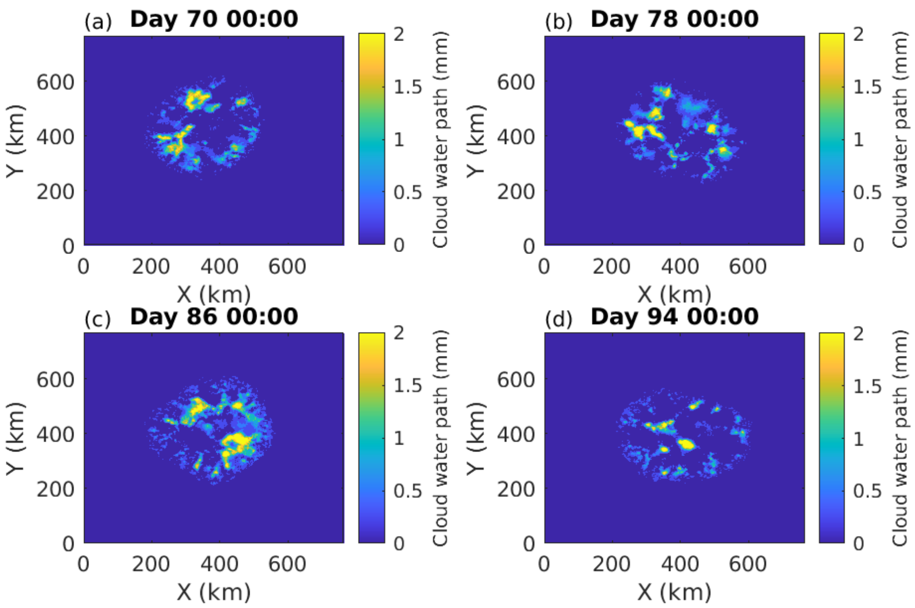

Figure S2. Snapshots of cloud water path (mm) for the AGG simulation at (a) day 70 00:00, (b) day 78 00:00, (c) day 86 00:00 and (d) day 94 00:00.

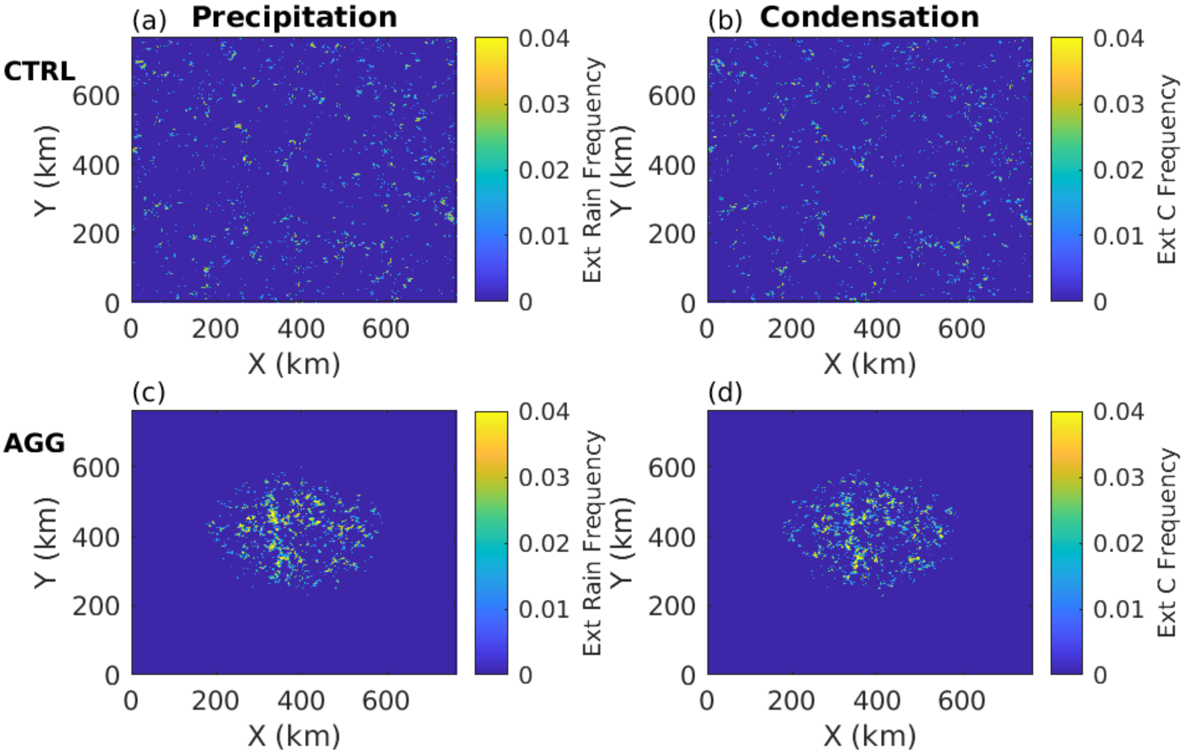

Figure S3. Occurrence frequency of extreme instantaneous precipitation (a,c) and extreme instantaneous condensation rates (b,d) for the CTRL (a,b) and the AGG (c,d) simulations. Extremes were defined as the 99th percentile of rainy events, and as the 99th percentile of the N most intense condensation rates, where N equals the number of rainy events.

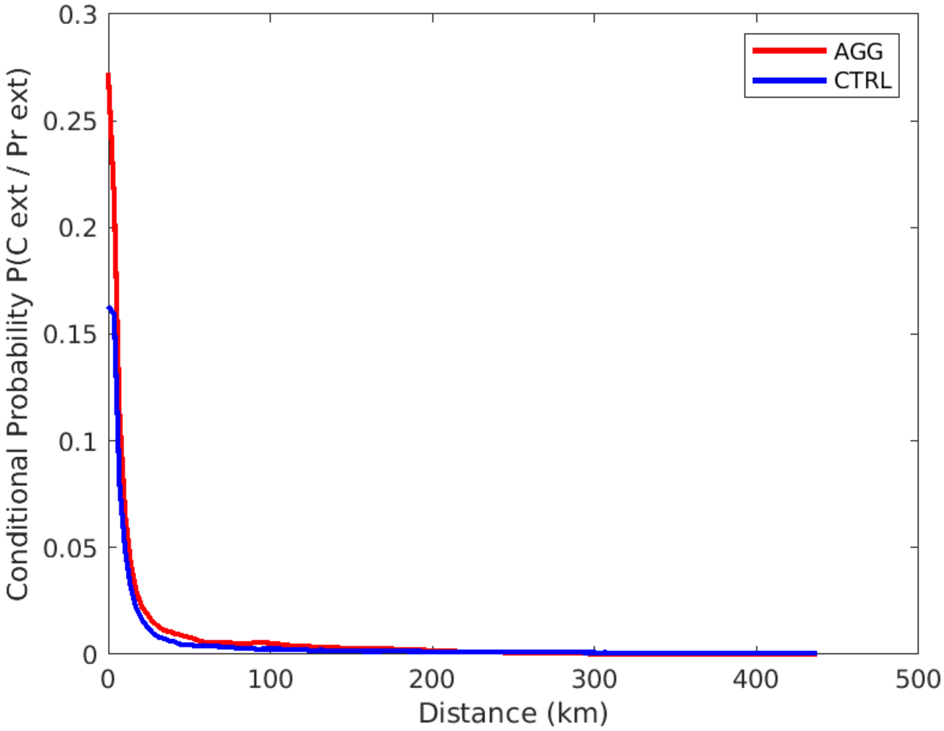

Figure S4. Conditional probability of extreme condensation rates occurrence knowing the occurrence of an extreme precipitation rate as a function of the distance from this extreme in precipitation for the CTRL (red) and the AGG (blue) simulations. Extremes were defined as the 99th percentile of rainy events, and as the 99th percentile of the N most intense condensation rates, where N equals the number of rainy events.

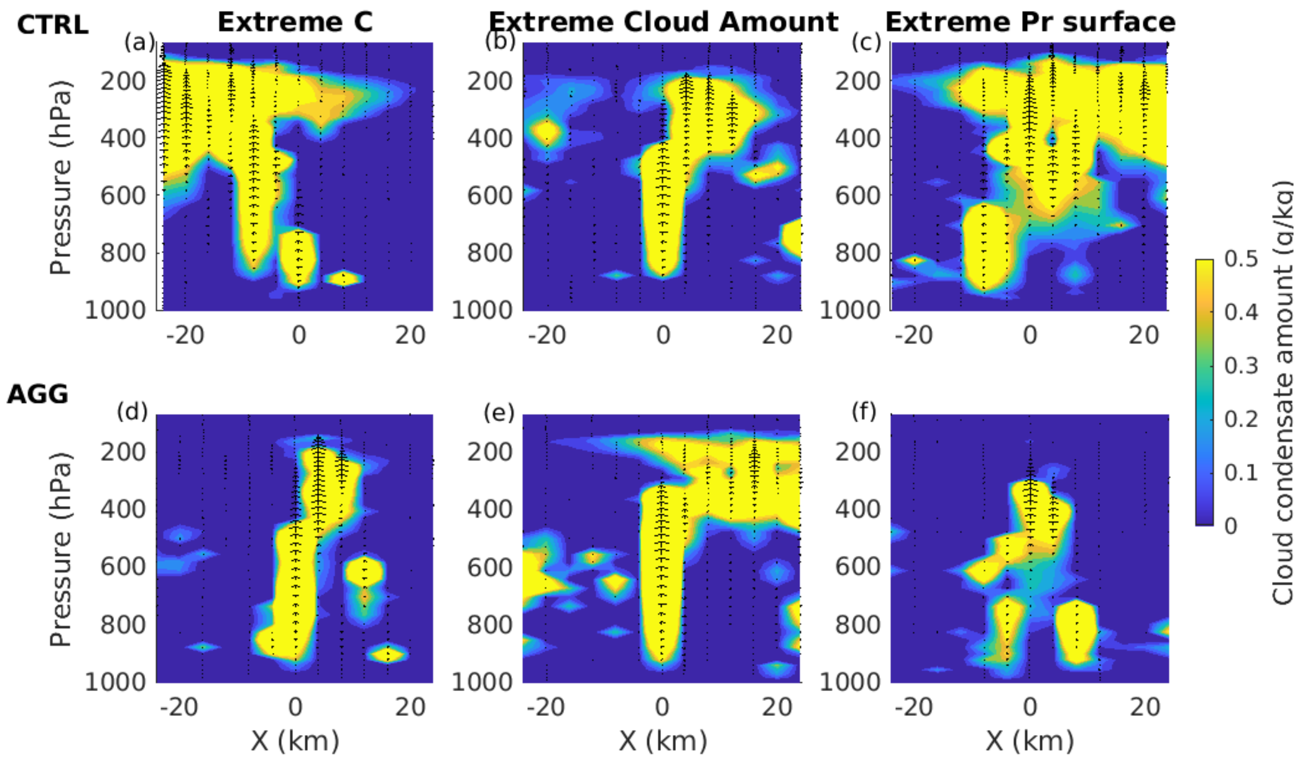

Figure S5. Composites of cloud condensate mixing ratio sections for individual events belonging to extremes condensation rates (C; a,d), cloud condensate mixing ratio (b,e), and surface precipitation (Pr; c,f) for the aggregated (a,b,c) and unorganized (d,e,f) simulations. Extremes are defined by events above the 99th percentile. Vertical arrows shows vertical velocity. The horizontal axis represents the distance from the grid point that experienced the extreme, and the vertical axis is in pressure coordinates.

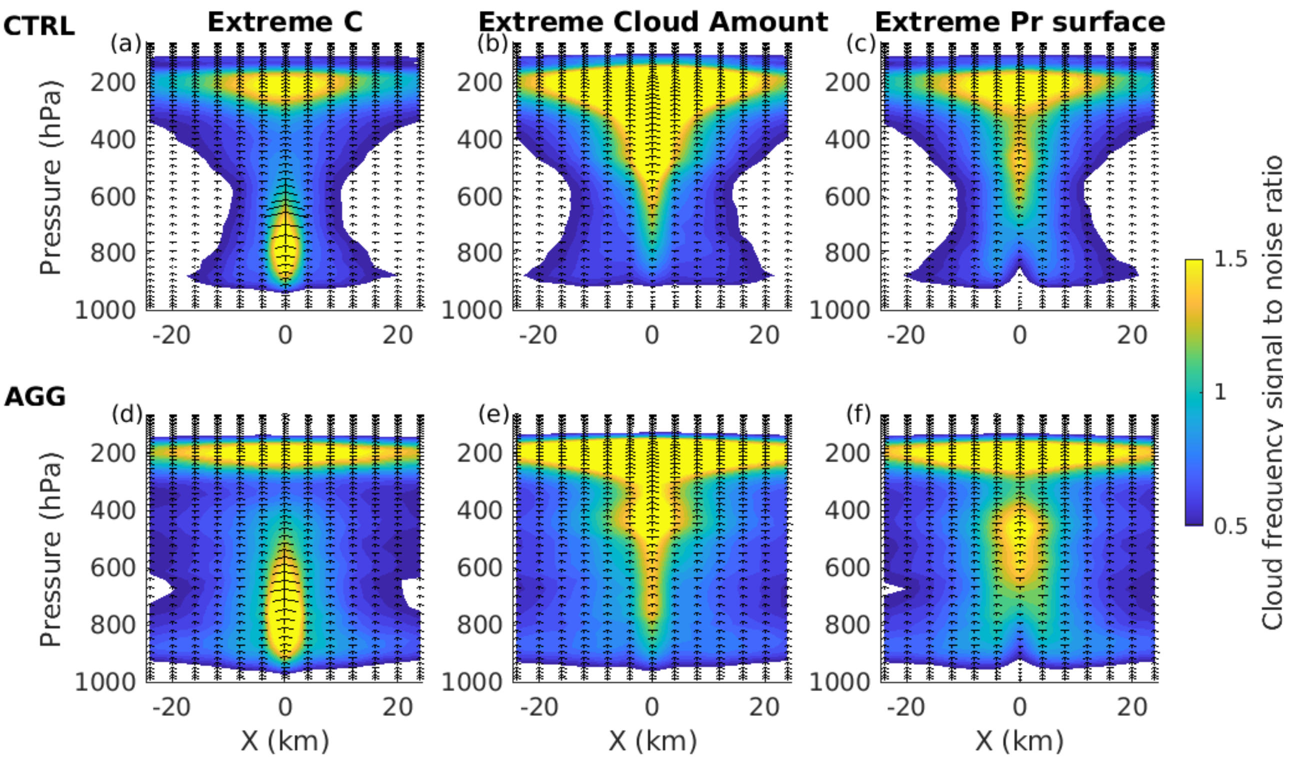

Figure S6. Composites of the signal to noise ratio of cloud frequency sections for extremes condensation rates (C; a,d), cloud condensate mixing ratio (b,e), and surface precipitation (Pr; c,f) for the aggregated (a,b,c) and unorganized (d,e,f) simulations. Extremes are defined by events above the 99th percentile. Vertical arrows shows the signal to noise ratio of positive vertical velocity frequency. The horizontal axis represents the distance from the grid point that experienced the extreme, and the vertical axis is in pressure coordinates.

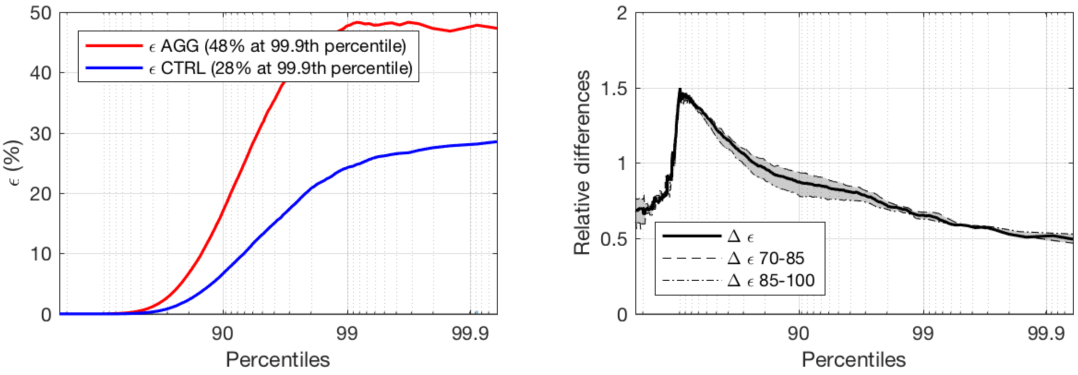

Figure S7. Precipitation efficiency for both AGG and CTRL simulations (left) and difference of precipitation efficiency between both simulations (right) as a function of percentile.
